# Age Affects the Expression of Maternal Care and Subsequent Behavioural Development of Offspring in a Precocial Bird

**DOI:** 10.1371/journal.pone.0036835

**Published:** 2012-05-11

**Authors:** Florent Pittet, Maud Coignard, Cécilia Houdelier, Marie-Annick Richard-Yris, Sophie Lumineau

**Affiliations:** UMR 6552 Ethologie animale et humaine, CNRS-Université de Rennes 1, Rennes, France; Université Pierre et Marie Curie, France

## Abstract

Variations of breeding success with age have been studied largely in iteroparous species and particularly in birds: survival of offspring increases with parental age until senescence. Nevertheless, these results are from observations of free-living individuals and therefore, it remains impossible to determine whether these variations result from parental investment or efficiency or both, and whether these variations occur during the prenatal or the postnatal stage or during both. Our study aimed first, to determine whether age had an impact on the expression of maternal breeding care by comparing inexperienced female birds of two different ages, and second, to define how these potential differences impact chicks’ growth and behavioural development. We made 22 2-month-old and 22 8-month-old female Japanese quail foster 1-day-old chicks. We observed their maternal behaviour until the chicks were 11 days old and then tested these chicks after separation from their mothers. Several behavioural tests estimated their fearfulness and their sociality. We observed first that a longer induction was required for young females to express maternal behaviour. Subsequently as many young females as elder females expressed maternal behaviour, but young females warmed chicks less, expressed less covering postures and rejected their chicks more. Chicks brooded by elder females presented higher growth rates and more fearfulness and sociality. Our results reveal that maternal investment increased with age independently of maternal experience, suggesting modification of hormone levels implied in maternal behaviour. Isolated effects of maternal experience should now be assessed in females of the same age. In addition, our results show, for first time in birds, that variations in maternal care directly induce important differences in the behavioural development of chicks. Finally, our results confirm that Japanese quail remains a great laboratory model of avian maternal behaviour and that the way we sample maternal behaviour is highly productive.

## Introduction

The influence of maternal behaviour on offspring behavioural and physiological development is well documented. This particular behaviour is determined by interactions between females’ past and present social and physical environments and endogenous factors. One of these latter factors, age, has been widely studied for its influence on the expression of iteroparous species’ maternal behaviour in a large range of scientific fields including psychology [1], [2], neuro-endocrinology [3], behavioural and evolution ecology [4]–[12]. Here we focus on age-related variations of post-natal maternal care.

Age has been reported to increase the breeding success of many iteroparous vertebrates [13]–[15]. Three main hypotheses attempt to explain this age-related increase of offspring survival [10]. Two of the hypotheses are based on increase of parental efficiency. The experience hypothesis [12] predicts that the older the parents are, the more skilled they are, accumulating care experience through previous breeding periods, or foraging skills throughout their lifetime. The selection hypothesis [12] predicts that age induces a progressive disappearance of low quality phenotypes, resulting in an increase of reproduction performance with age. The third hypothesis is based on increasing investment of parents: the effort hypothesis (or restraint hypothesis) predicts that older mothers make more effort because of a decrease of their residual reproductive value [11], [16]. These hypotheses are not mutually exclusive and all have been tested and supported by data 6,8,9,16. As breeding success has been estimated mainly for free-living birds, separation between effects of age and of experience is impossible. To investigate effects of age on maternal behaviour independently from maternal experience, the maternal behaviour of females of different ages but with the same breeding experience should be evaluated and compared, and this requires totally controlled conditions.

Breeding success is the best index of care quality, but variations of offspring survival with parents’ age are not related only to maternal postnatal care variations but also to genetic influences, prenatal influences and environmental influences. Few studies have reported an influence of maternal age on the expression of maternal care during the postnatal period and then they mainly concerned mammals. Activities such as suckle span length in red deer hind [13], protectiveness during the first 20 days post-birth in feral horses [6], time spent nursing and tolerance towards calves in bison [17], time spent in ventro-ventral contact in macaques [18], and to decrease protectiveness in this latter species have been reported to increase with age. Reports for birds are limited to foraging time by California gulls [7] and brood defence by red jungle fowl [19] that increase with age. However, data concerning qualitative and quantitative variations of different traits of avian maternal care during the postnatal period related to the influence of age are lacking, particularly data from experiments under standardized conditions that erase effects naturally associated with age such as accumulation of experience and prenatal influence on chicks.

The influence of mammalian maternal care variations on offspring subsequent behavioural characteristics have been largely studied [20]–[23]. Quantity of care by rodents influences offspring stress reactivity, defensive behaviour and reproduction strategies. Levels of rejection and protectiveness by primates influence their infants’ subsequent reactions to novelty and sociality. Recent studies report that mother birds strongly influence their offspring’s behavioural development. Indeed, maternal effects have been illustrated by large behavioural differences between brooded and artificially reared chicks [24] or through important non-genetic transmission of behavioural characteristics by adult females to their fostered chicks, concerning in particular their emotional reactivity [25] and their sociality [26]. However, maternal impacts on chicks’ behaviour have never been associated directly with characteristics of maternal behaviour expressed during the breeding period.

Moreover, effects of age on the general behaviour, on cognitive skills [27] and behavioural characteristics like emotional behaviour [28] or sociality [29] of mammals, particularly rodents, have been reported. We hypothesized that as age has an impact on non-brooding females’ behavioural characteristics, it should also impact the way females interact with their offspring during breeding, independently of the aforementioned theories. However, studies interested in the effects of age on maternal behaviour never estimate behavioural differences between mothers before the breeding period.

The aims of this study were threefold. The first aim was to compare the expression of various maternal behaviour traits during the postnatal phase between young and older female birds under controlled conditions; all these females were inexperienced so as to avoid effects of breeding experience. We then evaluated the influence of maternal behaviour variations on chicks’ weight and behavioural development. To make sure that potential differences in maternal behaviour were not associated to any age-related differences in other behavioural characteristics of the mothers, we evaluated both their sociality and their emotional reactivity before the breeding period.

Our subjects were naïve Japanese quail (*Coturnix coturnix japonica*) at two different ages: one set of 2-month-old females called “young” (hereafter Yg) and one set of 8-month-old females called elder (hereafter Ol). Observations in semi-natural environments indicate that typically only females of this species invest in parental behaviour [30]. Moreover, the fact that quail hens can foster chicks allows us to standardize prenatal influences that are known to vary with layers’ age, affecting both egg quality, chick weight at hatching and the future behaviour of offspring [31]. Behavioural tests first compared fearfulness and social motivation between 2- and 8-month-old inexperienced females. We then assessed their maternal behaviour during a whole breeding period and finally we compared the behaviour of the offspring brooded by these two sets of females.

Elder females spent longer warming chicks and were less rejective towards chicks. Both fearfulness and sociality differed significantly between the two sets of chicks during their behavioural development; these differences appeared related to differences in the care they received. The slight differences of mothers’ behavioural characteristics between the two sets observed before the breeding period cannot be at the root of differences in care; these differences consequently are directly related to maternal age.

## Materials and Methods

This study was approved by the regional ethics committee (agreement number: R-2011-SLU-02). Experiments were approved by the departmental direction of veterinary services (Ille et Vilaine, France, Permit number 005283) and were performed in accordance with the European Communities Council Directive of 24 November 1986 (86/609/EEC).

### Subjects and Housing

Our adult females and chicks came from a broiler line and were provided by an industrial farm: Les cailles de Chanteloup, Le petit Velobert, 35150 Corps-Nuds, France.

Adult females were divided into two sets: one set of 2-month-old females and one set of 8-month-old females. Twenty-two adult females of each set arrived at the laboratory when they were 3 weeks old, and were kept in batteries until they were placed, all at the same time, in wire-mesh cages (51×40×35 cm), in the same room where breeding occurred. Cages had opaque lateral walls and contained a feeder and a drinker. Food and water were available *ad libitum*. Temperature was 20±1°C and a 12∶12 light/dark cycle was maintained. Mean weight of elder females was higher than that of young females (Yg: 242.1±5.9 g; Ol: 295.6±8.8 g; Mann-Whitney U test: U = 63, p<0.001).

Chicks came from 320 eggs artificially incubated in our laboratory. Incubation lasted 17 days (37.7°C, 45–50% humidity). When chicks hatched, they were placed in groups of 40 in large plastic cages (98×35×42) equipped with a feeder, a drinker and a heater (38±1°C). They were placed with mothers to be fostered when they were one-day old. As morphological sexual dimorphism appears only at 3 weeks [32], chicks were randomly distributed to females of both set. Their sex was determined later but the sex ratios did not differ between sets (χ^2^ = 0.351, df = 1, p>0.05).

### Fostering Procedure and Observation of Maternal Behaviour

During the three weeks before the breeding period, mothers’ fearfulness and sociality were evaluated, then maternal behaviour was induced and observed. When chicks were 11 days old, the mothers were removed from the cages and chicks developed in sibling groups for 2 more weeks during which ethological tests evaluated their fearfulness and sociality.

**Figure 1:**
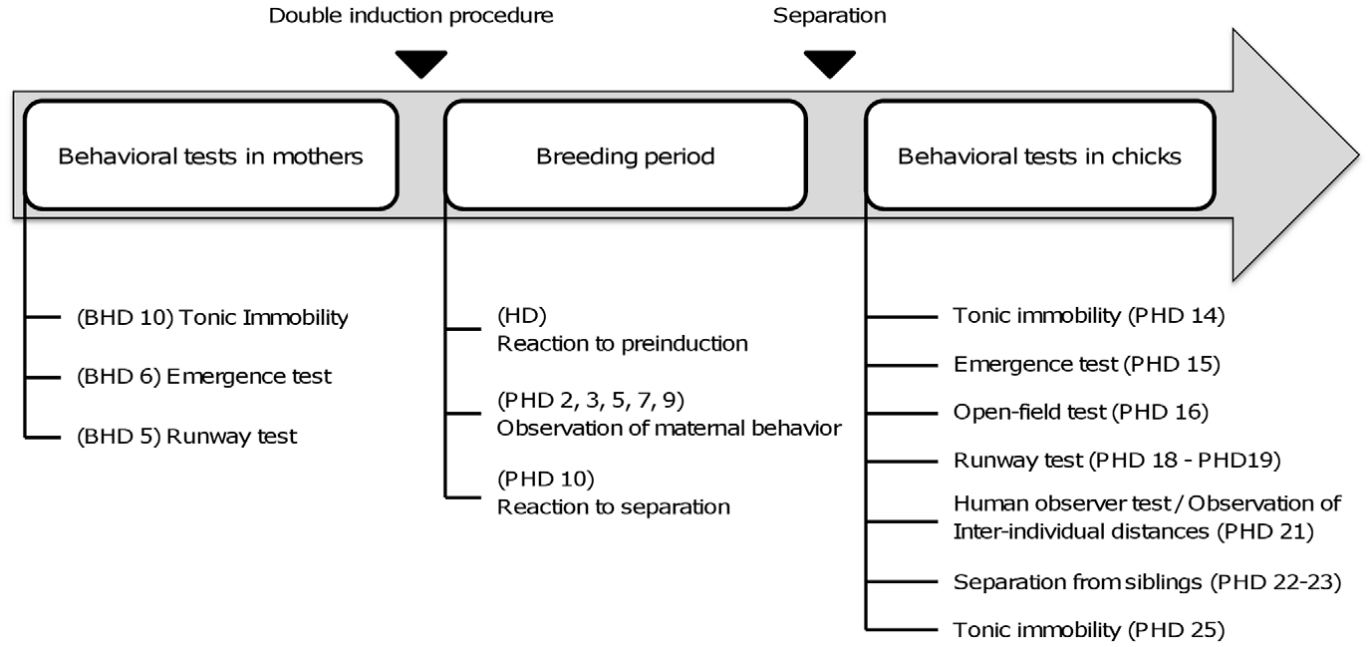
Experimental schedule. Test and observation schedule. Dates refer to hatching day of test chicks; (BHD: before hatching day, HD: Hatching day, PHD: post-hatching day).

Details of the temporal organization of the procedure are presented in figure 1.

#### Fostering and breeding period

Three weeks before the adult females of the two sets were respectively 2 and 8 months old, 22 females of each set were placed in the brooding cages to habituate to their environment. They were distributed so as two females of the same set were never in neighbouring cages. Maternal behaviour was then induced by a double induction procedure including pre-induction with two chicks and induction with four experimental chicks the following day. For details concerning induction of maternal behaviour see [33].

At the beginning of the dark phase, 2 one-day-old chicks were placed gently underneath each female who had been shut up in her nest box (18×18×18 cm) one hour before. Boxes were shut up again for the whole night during which the chicks’ vocal and physical solicitations induced rapid expression of maternal behaviour by the adult females. The next morning, all the boxes were opened and removed from the cages. Chicks that showed signs of hypothermia when leaving the boxes were replaced. During this first day that the mothers spent with this first brood, their maternal behaviour was recorded. At this stage, females that did not express any warming behaviour of the young chicks were excluded from the experience: thus four of the 22 Yg and four of the 22 Ol were excluded.

In the evening of this first day the mothers were closed-up again in their nest box for the whole night and the first two chicks were replaced by the four one-day-old test chicks that would then stay with the females for the whole breeding period. This double induction procedure ensures that all mothers are already maternal before having to foster the experimental chicks so that their influence on the chicks is not related to latency of expression of maternal behaviour. A total of 88 chicks were used for the pre-induction and 144 chicks were used for the induction. Chicks brooded by young females are noted C-Y and chicks brooded by elder females are noted C-O. As the natural brooding period lasts 11 days after hatching [34], we recorded the interactions between mothers and chicks from post-hatch day (hereafter PHD) PHD 2 to PHD 11 (fig. 1). Then, mothers were separated from chicks and chicks’ reactions to this separation were recorded. Chicks then remained with their siblings for two more weeks during which the fearfulness and the social motivation of two chicks from each cage were evaluated. Chicks were randomly chosen and sex ratios did not differ between C-Y and C-O (χ^2^ = 0.003, df = 1, p>0.05).

#### Observations of maternal behavior

Maternal behaviour was recorded on PHD 2, PHD 3, PHD 5, PHD 7 and PHD 9. We evaluated maternal behaviour using both instantaneous scans samplings to establish mother’s time-budgets, associated with focal animal sampling to note rare behaviours. The observer was placed behind a one-way mirror.

##### Instantaneous scan sampling

Each day we recorded 40 scans at 6-minute intervals: 20 scans in the morning and 20 in the afternoon. For each scan, we recorded whether the mother was warming chicks and if she was we recorded her posture and how many chicks were being warmed. We also recorded the distance between each chick and its mother and the mother’s activity. We recorded whether chicks were warming one another. The behavioural traits recorded are defined in table 1. Data were sampled using an ipod Touch (Apple©) and the application “scan sampling” (Vincent Richard ©).

**Table 1:** Parameters and definition of behavioural traits recorded during the scan sampling observation sessions.

Parameters		Definitions
**Warming activity**	Yes/no	Mother is motionless and at least one chick is partially or entirely covered by her feathers
**Warming posture**	Covering posture: Chick(s) is/are completely hiddenunder their mother’s feathers	Lying down: Both feet and tibio-tarsal articulations touch the floor, body, neck huddled up, touching the floor
		Crouched: Both feet and tibio-tarsal articulations touch the floor, body is slightly raised, head raised up, feathers touch the floor but the belly does not
		Medium: Feet touch the floor, but tibio-tarsal articulations do not and feathers are close to the floor
	Non-covering posture: Chicks are partially exposedto the environment	Lying on one side: The female is stretched out, her flank touches the floor, chicks must snuggle against her to be warmed
		High: The female is standing up, legs straight, her body is too high for the chicks to be completely covered
**Number of warmed chicks**	0/1/2/3/4	Number of chicks entirely or partially covered by mother’s feathers during a warming phase
**Maternal activity**	Rest/observe/feed/explore/self-preen/dust bathe/jump/alert/peck chick/preen chick/aggress chick	
**Chicks’ self warming**	yes/no	Chicks are huddled up against one another, motionless
**Distance chick-mother**	Under	Chick is under the female
	Close	Chick is not under the female but in contact with her
	Near	Chick is one chick length max from the female
	Far	Chick is between one chick length and half the cage away from the female
	Far away	Chick is between half the cage length and cage length
	Opposite	Mother against one cage wall and chick is against the opposite wall

##### Focal sampling

Each cage was observed for two 4-minute sessions. The entire behavioural sequence of the mother was recorded. We also recorded additional traits that included contact breaks between chicks and their mother, who initiated the break (mother/chick), posture changes and trampling chicks.

##### Separation test

When the chicks were 10 days old (PHD 10), they were taken out of their cage away from their mother and the reactions of each mother was recorded during a 5-minute focal sampling. These data yielded the latency and frequencies of distress calls, of comfort behaviours such as resting or eating and the frequencies of all other behaviours.

### Behavioural Characteristics of Foster Mothers and Chicks

Behavioural tests assessed levels of fearfulness and sociality of both mothers and chicks. Mothers were tested during their phase of habituation to their breeding environment. Chicks were tested after separation from their mothers (fig. 1). All observations except the reactions to humans were recorded behind a one-way mirror.

#### Fearfulness tests

##### Tonic immobility test

This test followed the protocol described by Jones [35]. Tonic immobility (T.I.) is a reflexive response to a fear-inducing stimulus and response duration is positively correlated with fearfulness. Each test chick was removed from its cage and placed on its back in a U-shaped wooden cradle and held in this position for 10 seconds prior to release. The experimenter, placed out of the sight of the subject, recorded both number of induction(s) required to obtain a T.I. duration lasting at least 10 seconds, with a maximum of 5 inductions, and the duration of tonic immobility, with a maximum of 300 s. Zero s was scored when the subject never remained in T.I. for 10 seconds.

##### Emergence test

Each test individual was removed from its home cage and transported in the dark, in a wooden box (18×18×18 cm). This box was then placed on the left side of the apparatus: a large and well-lighted wooden box (62×60×33 cm) with wood-shavings covering the floor and an observation window. When the transport box was placed in the apparatus, it was kept closed for 1 minute and the latency of the first distress call and the numbers of calls emitted by the chick were recorded. Then, the door was left open for 3 minutes. Latencies to pass its head out of the box and to emerge completely were recorded. These data give a good estimate of fearfulness as emergence latency and animal fearfulness are positively correlated [36], [37]. Once the animal is in the test cage, the transport box is closed and the chick is observed for 3 minutes. The latency of its first distress call, the number of distress calls and the frequency of exploration, observation, locomotion and maintenance activities were recorded.

##### Openfield test

Chicks were placed individually in the centre of a wire netting arena (Ø120×60 cm) with a linoleum floor, in the dark. Then the light was switched on, and, hidden behind a one-way mirror, the experimenter recorded the latency of the first distress call, the number of distress calls, the latency of the first step, the number of steps and the frequency of observation, exploration and maintenance activities.

##### Human observer test

Quail were tested in their familiar environment. The experimenter, using instantaneous scan sampling, passed in front of each cage at 5-minute intervals recording a total of 32 scans for each cage. Each time he passed in front of a cage, he stopped for few seconds and recorded the reactions of all individuals: fear reactions (individual interrupts its activity and moves away from the observer), observation of the observer, or no reaction to his presence.

#### Sociality tests

Emergence and open-field tests confront subjects with both a novel environment and social isolation. Some activities recorded during these tests are associated with social motivation such as distress calls and jumps [38].

##### Separation from siblings

Each chick was removed from its home cage and placed alone in a similar cage for 3 minutes. The latency of first distress call and first step and the numbers of distress calls and of steps were recorded.

##### Inter-individual distances

When they were 21 days old, inter-individual distances between each chick and its nearest conspecific were recorded by 32 scans of each cage, made at 4-minute intervals. Distance classes were the same as those used to describe distance between each chick and its mother during the brooding period (Table 1).

An index of distance was calculated using the following formula:

Index of distance = (N OPPOSITE+N FAR AWAY×0.75+N FAR×0.5+N NEAR×0.25)/(Total N scans).

##### Runway test

This test is an adaptation of the treadmill test [39] that allows test individuals to reach a social stimulus [26]. The apparatus is a 100 cm-long wire-netting tunnel. Test individuals were transported in a wooden box (18×18×18 cm), which was then placed at the tunnel entrance. At the other end of the tunnel was a cage (20×35×20 cm) containing three unfamiliar chicks of same age, representing a social stimulus. The corridor was divided into four zones: the closest zone to the social stimulus, “1 bird long”, was named proximal zone or Zone P. The rest of the length was divided into 3 equal 32 cm-long zones; they were called, from the entrance to the zone P: zones A (beginning of the tunnel), B (middle) and C (end of the tunnel). One minute after the transport box had been placed at the entrance, it was opened and the time a chick took to emerge was recorded. Once the chick was in the corridor, the box door was closed and, for 5 minutes, an observer recorded time to reach zone P, time spent in each zone, frequency of exploration of the cage containing conspecifics, aggressions, fear postures, and jumps.

### Statistical Analysis

As most of our data were not normally distributed, we used non-parametric statistical tests to compare behavioural expressions between Yg and Ol females and between C-Y and C-O. Mann-Whitney tests compared frequencies, latencies and proportions of time between Yg and Ol and between C-Y and C-O. Chi-square tests compared proportions of animals of each set. All data analyses were computed using statistica®.

## Results

### Mothers’ Behaviour Tests

All females remained in tonic immobility after the first induction attempt for more than 10 seconds and T.I. durations did not differ significantly between Yg and Ol (Mann-Whitney U-test: p>0.05).

Vocalizations were rarely emitted in the emergence and the runway tests, so they were not included in the analysis.

Latencies to leave the box in the emergence test did not differ significantly between the two sets of females (Mann-Whitney U-test: p>0.05). However, Yg females explored the wood shavings more frequently (Yg: 9.61±1.16; Ol: 5.86±1.04, Mann-Whitney U-test: U = 152.5, p = 0.023) and dust-bathed more (Yg: 2.13±0.80; Ol: 0.41±0.299; Mann-Whitney U-test, U = 167.5, p = 0.013) than did Ol females. Frequencies of locomotion, observation or maintenance behaviours did not differ significantly between Yg and Ol females (Mann-Whitney U-test: p>0.05).

Latencies to leave the box or latencies to reach the social stimulus in the runway test did not differ significantly between the two sets of females (Mann-Whitney U-test: p>0.05). Aggressions, jumps and fear postures were never expressed during this test. Neither latencies and frequencies of distress calls nor frequencies of exploration of the conspecifics’ cage differed between the two sets of females in the runway test. Females spent similar proportions of time in the non-social zone (zone A) and in the social zone (zone P), but Yg females spent significantly more time in the medium zones (zone B: Yg: 42.49±14.56 s.; Ol: 8.031±2.15 s.; Mann-Whitney U-test: U = 147.5, p = 0.0168; Zone C: Yg: 44.46±7.93 s; Ol: 15.91±3.85 s.; Mann-Whitney U-test: 135.5, p = 0.008).

### Maternal Behaviour

#### Laying Behaviour

During the 3 days before the breeding period, mean numbers of eggs laid did not differ between Yg and Ol mothers (Yg: 1.5±0.3; Ol: 0.9±0.2; Mann-Whitney U-test: U = 315, p = 0.14). However, Yg laid more eggs during the breeding period (Yg: 4.4±0.5, Ol: 3.1±0.5, Mann-Whitney U-test: U = 239.5, p = 0.034).

#### Reactions to pre-induction

When opening the boxes on the first morning after addition of chicks, more chicks that spent the night with a young female than chicks that spent the night with elder females showed signs of hypothermia (closed eyes, trembling, motionless) and had to be replaced (chicks with Yg female: 0.522±0.176; chicks with Ol females: 0.045±0.045; Mann-Whitney U test: U = 158, p = 0.021). Subsequently after replacement, that same day, frequencies of chick-aggression by females and numbers of chicks showing signs of hypothermia did not differ between the two sets (Mann-Whitney U-test: p>0.05).

After the induction night, numbers of C-Y and C-O chicks that showed signs of hypothermia when opening the boxes did not differ significantly (Mann-Whitney U-test: p>0.05).

#### Warming parameters

**Figure 2:**
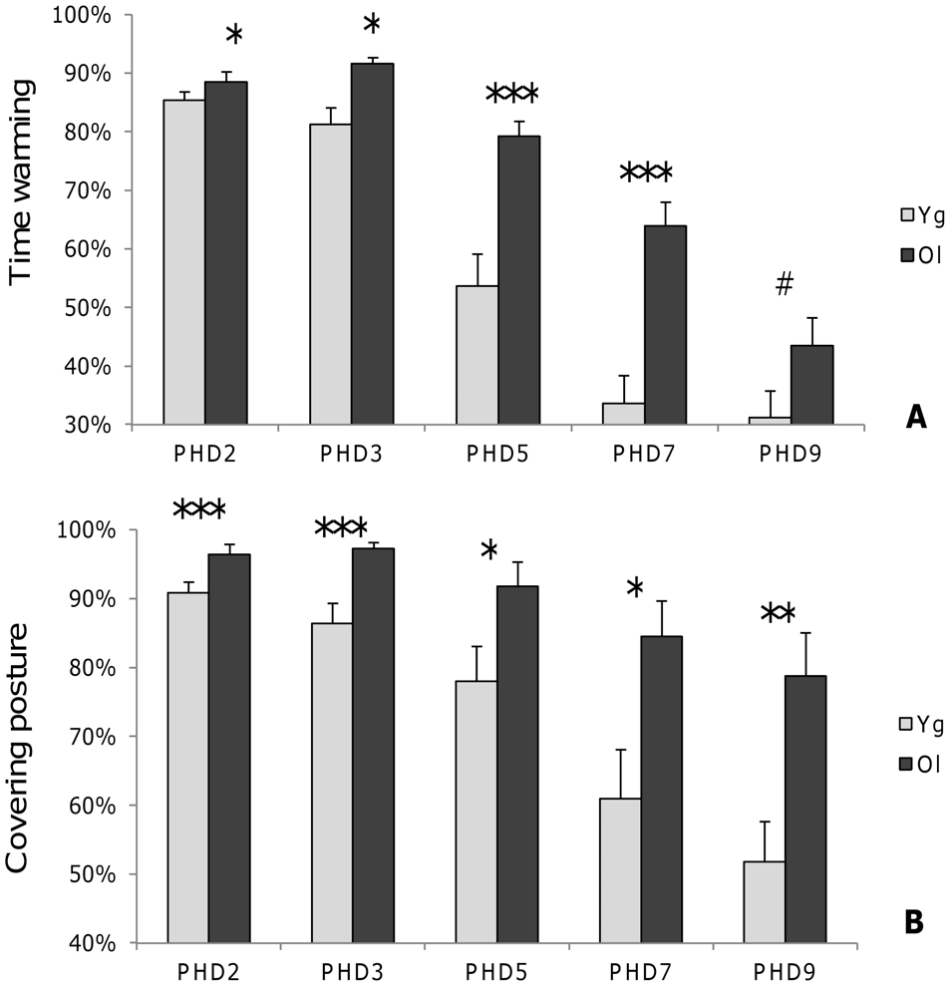
Warming and covering posture rates. Time spent warming (A) and time spent warming in covering posture (B) for the five maternal behaviour observation days (mean ± SE percentage). Mann-Whitney U-test #0.1>p,*: 0.05>p, **: 0.01>p, ***: p<0.001.

Results are presented in table 2. Elder females spent more time warming their chicks from the beginning and until PHD 7 (fig. 2-A). Analysis revealed significant differences in warming posture preferences and, more particularly, that elder females presented covering postures more frequently (fig. 2-B). Elder females warmed more chicks during a warming period, than did young females, from PHD 3 to PHD 9. Young females changed posture most frequently on PHD 2, 3 and 5 and expressed a greater variety of postures on these days.

**Table 2:** Behaviours of young and older females during breeding period.

		day 2			day 3			day 5			day 7			day 9		
Category	Parameter	young ♀	*p*	elder ♀	young ♀	*p*	elder ♀	young ♀	*p*	elder ♀	young ♀	*p*	elder ♀	young ♀	*p*	elder ♀
Warming	Time warming (%)	85,1±1,4	*	**88,5±1,7**	81,8±2,9	*	**91,6±1,1**	53,6±5,5	***	**79,2±2,6**	33,6±4,8	***	**63,9±4,1**	31,2±4,6	#	43,5±4,8
	Covering postures whenwarming (%)	90,8±1,6	***	**96,4±1,5**	86,4±2,9	***	**97,3±0,9**	78,0±5,1	*	**91,8±3,5**	61,0±7,1	*	**84,5±5,2**	51,8±5,9	**	**78,8±6,3**
	Posture changes (frequency)	**6,5±0,9**	**	3,0±0,6	5,9±0,9	*	3,0±0,6	5,4±0,9	–	3,9±0,6	2,7±0,6	–	3,4±0,9	3,7±0,6	–	2,8±0,6
	Posture variety (number 1–5)	**3,9±0,2**	**	3,0±0,2	**3,8±0,2**	**	2,9±0,2	3,5±0,2	–	3±0,2	3,0±0,3	–	3,3±0,2	2,8±0,2	–	3,1±0,3
	Chicks warmed during warmingperiods (number 1–4)	3,2±0,05	–	3,3±0,06	3,2±0,1	*	**3,5±0,06**	2,7±0,2	*	**3,1±0,1**	2,3±0,2	*	**2,7±0,1**	2,2±0,2	–	2,3±0,1
Warming break	Break initiated bymother	**3,76±0,6**	**	1,6±0,39	**3,4±0,5**	**	1,2±0,22	2,79±0,52	–	2,4±0,3	1,26±0,34	–	1,6±0,39	1,32±0,31	–	1,11±0,21
	Break initiated by chick	1,05±0,22	#	1,85±0,33	0,52±0,19	–	1,05±0,36	0,11±0,07	***	**0,9±0,2**	0,11±0,07	**	**0,61±0,18**	0,11±0,07	*	**0,67±0,24**
	Proportion of breaks initiated by mother (%)	**71,1±7,1**	*	43,6±8,3	81,9±6,7	#	61,4±9	**97,8±1,5**	***	74,6±6,2	**89,6±8,4**	*	68,4±9,3	96,3±2,5	#	69±9,9
Abusive behaviours	Aggression (Fq)	0,05±0,05	–	0	0,05±0,05	–	0	0	–	0,15±0,15	0	–	0	0	–	0,06±0,06
	Chick trampling (Fq)	**1,43±0,62**	**	0	0,24±0,12	–	0,05±0,05	0,42±0,26	–	0,1±0,1	1,74±0,27	–	2,61±0,5	1,58±0,27	–	2,3±0,4
Activity	Observing (%)	31,8±3,3	**	**45,9±3,3**	32,3±2,2	***	**49,2±3,3**	28,8±2,7	**	**40,9±2,9**	27,5±2,3	**	**41,3±4**	27,6±2,9	**	**40±3,3**
	Resting (%)	9,5±1,6	*	**16,9±2,6**	21,4±3,5	-	22,4±2,2	10,9±2,4	***	**22,4±2,6**	9,9±1,6	#	18,5±3,7	11,4±2,1	–	13,6±2,8
	Feeding (%)	**29±1,8**	**	16,6±1,9	18,5±2,2	–	14,2±2	17,2±1,8	–	16,8±1,5	18±1,7	–	15,3±1,3	19,5±2	–	18,2±2,9
	Preening (%)	**12,6±1,4**	*	7,9±0,9	**12,7±1**	***	6,8±1	**14,2±1,6**	**	7,9±0,9	10±1,1	–	8,8±1,4	8,4±1,2	–	8,9±0,9
	Exploring (%)	6,7±1,2	–	4,8±1,2	7,3±1,6	–	4,3±0,8	**17,1±3,2**	**	6±1,2	**21,7±2,3**	**	10,6±2,4	**21,4±2,1**	**	11,4±2,3
	Alertness (%)	7,1±0,4	–	4±0,2	6,8±1,4	–	2,2±0,2	**9,5±1,1**	**	2,9±0,3	**10,9±0,6**	**	4,4±0,2	**9,3±0,5**	*	6,7±0,9
Distance of chicks andthermoregulation strategies	Under the mother (%)	65,1±0,8	*	**71,8±1**	57,6±3,5	**	**73,1±1,9**	33,6±5,1	***	**60,3±3,7**	16,9±4,2	**	**38,9±3,6**	11,2±2,7	**	**20,6±3,5**
	Close to the mother (%)	**17,4±0,5**	*	13,5±1,9	**16,2±2,1**	**	8,4±1	15,7±1,6	#	12,8±2,1	16,7±1,3	–	18,1±2	18±2	–	20,2±1,8
	Near the mother (%)	**4,3±0,5**	*	3,1±0,4	3,4±0,4	–	2,6±0,3	**7,3±0,9**	*	4,1±0,5	9,9±0,9	#	7,5±0,8	10,9±1,3	–	9,7±1,2
	Far from the mother (%)	7,7±0,8	–	6,4±0,6	9,9±1,6	–	5,9±0,7	**24,2±3,1**	**	12,7±1,5	**28,4±2,1**	**	20±2	26,8±2	–	24,3±2,2
	Far away from the mother (%)	5,3±0,9	–	4,8±0,7	7,7±1,4	–	4,9±0,8	**18,1±2,4**	**	9±1,4	**25,9±2,1**	***	14,1±1,5	27,2±2,7	–	23,4±2
	Opposite (%)	0,2±0,1	–	0,3±0,2	0,1±0,1	–	0,1±0,1	1,2±0,5	–	1,1±0,5	2,2±0,4	–	1,5±0,3	**5,9±1,3**	**	1,8±0,4
	Self Warming (%)	–	–	–	0,1±0,1	**	**5,6±2,9**	6,4±1,8	***	**27,5±5,3**	14±2,7	**	**38,8±4,6**	29,8±4	*	**45,5±4,4**

Frequencies and percentages of behaviours (mean ± S.E). Bold values: significant, Mann-Whitney U-test, #: 0.1>p, *: 0.05>p, **: 0.01>p, ***: p<0.001.Frequencies of initiation of warming breaks differed between sets of females. Yg initiated warming breaks with chicks more frequently than did Ol females on PHD 2 and 3. C-O initiated more warming breaks on PHD 5, 7 and 9 than did C-Y. Proportionally more warming breaks were initiated by Yg mothers than by Ol mothers on PHD 2, 5 and 7 and tended to be higher on PHD 3 and 9.

#### Abusive behaviour and time-budget

Results are presented in table 2. Age of mothers also affects the expression of interactive behaviours and time budgets. Aggressions were rarely observed and their frequencies never differed significantly between Yg and Ol. On PHD 2, Ol never trampled their chicks, whereas a few Yg females were observed trampling. On other days trampling frequencies did not differ between the two sets of females. Time-budgets also differed during the brooding period between the two sets of mothers. Ol females spent more time in low energy-cost activities. Indeed, they spent more time observing on all 5 observation days. They also spent more time resting on PHD 2, 5 and tended to, on PHD 7. On the contrary, Yg females spent more time eating on PHD 2, exploring on PHD 5, 7 and 9, preening on PHD 2, 3 and 5 and tended to on PHD 7. Yg females spent more time alert from PHD 5 to 9.

#### Distance of chicks and thermoregulation strategies

Results are presented in table 2. Distances from their mother and strategies to get warmed differed significantly between C-Y and C-O.

C-O spent more time under the mother, from PHD 2 to PHD 9. C-Y spent more time close to the mothers on the first days after hatching, going further and further away from their mother as they grew older (PHD 5 and PHD 7), and they finally spent more time in the part of the cage opposite to their mothers on PHD 9 than did C-O.

As absolute distance of chicks from mother was closely linked to the time the mother spent warming them, we analysed the relative distances between chicks and mother, i.e. their distance from her when they were not being warmed. No differences between the two sets could be evidenced until PHD 7 when elder females’ chicks spent significantly more time close to the female (relative rate of time close to the female: C-Y: 21.4±2.4%; C-O: 30.3±3.2%; Mann-Whitney U-test: U = 88; p = 0.012) and conversely C-Y spent significantly more time far from their mother (relative rate of time far from the female: C-Y: 34.1±2.5%; C-O: 24.6±2.7%; Mann-Whitney U-test: U = 93, p = 0.020). On PHD 9, when chicks were not being warmed, C-Y spent more time in the part of the cage opposite to their mother than did C-O (relative rate of time in the opposite part of the cage: C-Y: 6.6±1.4%; C-O: 2.3±0.6%; Mann-Whitney U-test: U = 94, p = 0.020).

Neither C-Y nor C-O warmed one another on PHD 2. Later, C-Y spent more time warming one another from PHD 3 and until PHD 9.

#### Reaction to separation

When separated from their chicks, Yg females presented shorter feeding latencies (Yg: 55.8±16.9; Ol: 184.4±26.42; Mann-Whitney U-Test: U = 60; p<0.001), more feeding bouts (Yg: 10±2; Ol: 4.3±1.1, U = 91, p = 0.015) and more floor explorations (Yg: 2.84±0.75, Ol: 0.89±0.43; Mann-Whitney U-test: U = 90, p = 0.008).

### Chicks’ Growth and Behavioural Characteristics after Separation

#### Chicks’ weight

When they were separated from their mothers on PHD 11, chicks’ weights did not differ significantly between the two sets (C-Y: 44.20±1.40; C-O: 46.40±1.26; Mann-Whitney U-test: p>0.05). Later, 25-day-old C-O were heavier than C-Y (C-Y: 134.67±4.17 g; C-O: 146.51±2.97 g; Mann-Whitney U-test: U = 1420, p = 0.046).

#### Chick’s fearfulness

##### Tonic Immobility test

Tonic immobility durations did not differ significantly between the two sets of chicks, neither on PHD 14 nor on PHD 25 (Mann-Whitney U-test: p>0.05). On average more induction attempts were required to obtain a 10 s. T.I. duration in chicks brooded by elder females than in C-Y (C-Y: 1±0; C-O: 1.11±0.05; Mann-Whitney U-test: U = 592, p = 0.04). However this difference is due only to the fact that four chicks brooded by elder females required two attempts to induce T.I., whereas T.I. following the first induction for all the other chicks lasted more than 10 seconds.

##### Emergence test

Chicks brooded by elder females emerged later (C-Y: 4.4±0.7 s; C-O: 7.6±1.3 s; U = 486.5, p = 0.046). No other significant difference could be evidenced between the two sets of chicks (Mann-Whitney U-test: p>0.05).

##### Open-field test

Chicks brooded by elder females emitted more distress calls (C-Y: 79.9±10.0; C-O: 119.0±12.4; Mann-Whitney U-test: U = 452, p = 0.018) and explored the walls of the apparatus more often (C-Y: 0.8±0.3; C-O: 2.7±0.7; Mann-Whitney U-test: U = 498.5, p = 0.031).

##### Human observer test

**Figure 3:**
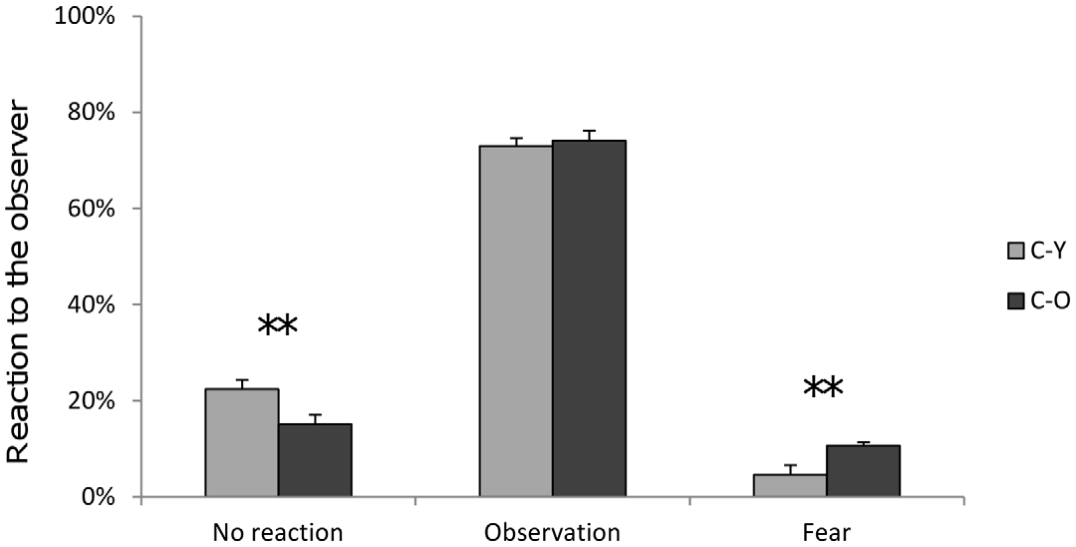
Reaction to humans. Behaviour expressed by C-Y and C-O in reaction to humans (mean ± S.E. %). Mann-Whitney U-test **: 0.01>p.

When they perceived the observer, both C-Y and C-O chicks responded with high rates of observation, and these rates did not differ significantly between the two sets (Mann-Whitney U-test: p>0.05). Nevertheless, C-Y did not react to the observer more frequently whereas C-O chicks showed higher rates of fear (fig. 3).

#### Chick’s sociality

##### Separation from siblings

Following separation from siblings, C-Y took more steps (C-Y: 140.59±20.26; C-O: 79.83±10.87; Mann-Whitney U-test: U = 485.5, p = 0.046), explored the wood shavings more (C-Y: 3.57±0.7; C-O: 1.5±0.37; Mann-Whitney U-test: U = 417, p = 0.005) and expressed more self-preening (C-Y: 1.21±0.3; C-O: 0.58±0.2; Mann-Whitney U-test: U = 493.5, p = 0.034). Chicks brooded by elder females tended to emit more distress calls (C-Y: 69.9±9.2; C-O: 107.7±14.1; Mann-Whitney U-test: U = 501.5, p = 0.07).

##### Inter-individual distances

Mean distances of chicks to their nearest conspecific did not differ significantly between the two sets of chicks (Mann-Whitney U-test: p>0.05).

##### Runway test

Chicks brooded by elder females took longer to emerge (C-Y: 2.9±0.8 s; C-O: 21.1±7.5 s; Mann-Whitney U-test: U = 448, p = 0.01) and to reach the social zone (C-Y: 32.8±12.5 s; C-O: 59.9±16.7 s; Mann-Whitney U-test: U = 486, p = 0.046). Neither times spent in each zone nor behaviour in this test differed significantly between the two sets of chicks (Mann-Whitney U-test: p>0.05).

## Discussion

Young and elder females differed from their first encounter with chicks. Indeed, more chicks that spent the pre-induction night with young females showed signs of hypothermia the following morning, suggesting that young mothers had not accepted the chicks during this first night as easily as elder females had. As we found no other significant differences in chicks’ states later, our results suggest that young females become maternal only after a longer latency. After induction, when females are maternal, elder mothers warmed chicks for longer, presented more covering postures and broke contact with chicks less frequently than did young females who behaved abusively and trampled chicks during the first day following induction. Finally, time-budgets differed significantly between the two sets of females as elder females presented higher rates of motionless activities like observation or resting.

Differences in maternal behaviour could be either a direct effect of maternal age or a consequence of differences in non-maternal behavioural characteristics impacting the way they care for their offspring. Indeed, age has sometimes been reported to affect such behavioural characteristics directly, particularly by increasing rodents’ anxiety and decreasing their social interactions [28], [29], [40]. Moreover, we know that these behavioural characteristics, i.e. fearfulness and social motivation, are transmitted from Japanese quail hens to their fostered chicks, suggesting that they may impact the way hens breed chicks. The quality of young females’ first interactions with chicks, during pre-induction, may have been a byproduct of their greater fearfulness, as neophobia is known to alter the first interactions between mother and offspring in another precocious species [41]. Nevertheless, our data concerning both sociality and fearfulness before the breeding period evidenced no significant differences between the two sets of females. Moreover, we found that young females expressed slightly more comfort behaviours in unfamiliar environments such as more dust-bathing in the emergence test and more moving along the corridor in the runway test. This could be a sign of a slight difference in anxiety levels as in rats [28], and confirms that the first interactions with chicks are not the result of differences in females’ neophobia levels, but are rather directly related to age-related differences in the facility to induce maternal behaviour. We also questioned whether differences in time-budgets were directly related to maternal investment, or to differences in basic activity levels between young and elder females, as young females expressed more energy-costly activities. Actually, data from behavioural tests before the breeding period evidenced no differences in global activity between young and elder females. Moreover, when analysing a low energy-cost activity that could interfere with warming for instance, young females self-preened at higher rates, suggesting that these time-budget differences are more a consequence of the time females spent warming chicks.

Our results may be interpreted according to the effort hypothesis as a greater maternal investment by elder females. Our laying behaviour data also support this hypothesis as they evidence that elder females resumed laying after induction of maternal behaviour later than did young females, this suggests that young females may invest less time caring for their offspring and that they would be ready sooner to initiate another breeding cycle. Compared to these ultimate factors, proximate factors influencing age-related maternal have received less attention. Richard-Yris et al. [42] found no differences in concentrations of testosterone, estradiol, androstenedione or prolactin before induction of maternal behaviour between two sets of mature domestic hens differing only by their age. These authors focused mainly on the females’ state of maturity and the age difference was probably too small to reveal variations in hormones implied in maternal behaviour. Prolactin is one of the main hormones focused when studying maternal investment in birds. The concentration of this hormone increases with age until senescence in a long-live bird, the black-browed albatross [5]. Recently, plasma prolactin concentrations of a precocial bird were reported to be correlated with post-hatching maternal care and to increase following a sigmoid function [43], suggesting that maternal care is stimulated only when plasma prolactin reaches a threshold. If our young females had lower baseline prolactin concentrations, this could explain why it was harder to induce maternal behaviour in this set during the pre-induction phase. The tactile and auditory stimulations young females perceived during the night may have been insufficient to elicit this threshold, whereas later tactile, auditory plus visual stimulations were sufficient to induce maternal behaviour. Subsequently, neuro-physiological studies are required to determine whether these age-related changes in latency to become maternal and in maternal investment result from differences in baseline prolactin concentrations or in differences in the quantities of prolactin receptors in specific brain areas, as in rodents [3].

Our results concerning young females’ maternal performances can be interpreted as a greater difficulty for them to switch from laying to maternal behaviour, thereby affecting the way they care for their young during the whole breeding period. Nevertheless, as many young as elder females became maternal after induction, and behaved maternally appropriately to ensure chick survival although young females resumed laying during the breeding period. Consequently, although first interactions with chicks may differ slightly between young and elder females during the nocturnal induction, their ability to become maternal did not differ. As our observations were limited to females that became maternal after the first induction, differences in maternal care cannot be linked to differences in the efficiency of our induction procedure.

The maternal behaviour differences between our two sets of females had an observable effect on chicks from the beginning of the breeding period. First, as an obvious consequence of differences in the time females spent warming them, chick-female distances differed. During all the breeding period, C-O spent longer under their mothers, whereas C-Y stayed close to their mother on PHD 1, then spent longer at medium distances from her and were finally observed more frequently the furthest away from their mother. This result could be linked to differences in chicks’ thermoregulation strategies according to maternal tolerance. Indeed, on PHD 1, C-Y spent longer close to their mother as they were trying to get warm even when the female refused. Later, they could move further away as they adapted to this refusal, regulating their thermoregulation by warming one another. Our results concerning relative chick-female distances show that during the first days, C-Y and C-O were at approximately the same distances from their mothers when they were not being warmed, confirming that differences in absolute distances are related to the time they can be actively warmed by mothers. Differences in relative distances appear from PHD 7 to PHD 9 when C-Y spent longer further from their mothers than did C-O. These results indicate that C-Y are more independent during the last days of the breeding period, suggesting that they could become emancipated earlier than C-O. Naturally occurring variations of primate offspring emancipation can be related to the maternal behaviour they received; higher rates of rejection are known to promote independence of infant Japanese macaques [23]. Our results reveal a similar trend. Our results concerning mothers’ reactions to separation from chicks support this hypothesis as elder females were more unsettled by this separation.

Chick weights did not differ between sets immediately after separation from their mothers, but later, when they were 25 days old, C-O were heavier than C-Y. We suggest that growth of C-Y was already delayed during the breeding period because of poorer warming conditions, known to influence growth rate in this species [44]. Authors indicate that chicks have to balance locomotion, thermoregulation and growth. In our case, C-Y probably invested more in locomotion during their first post-hatch days to follow rejective mothers. Later, as they were warmed less by mothers and spent more time warming one another, they warmed less efficiently, were more exposed to the cold, a fact that has been reported to increase metabolism, decrease digestive efficiency and therefore restrict growth.

Both emotional reactivity and social motivation differed between the two sets of chicks. The social and emotional reactivity of chicks brooded by elder females was higher. Fear-eliciting situations, such as reaction to humans, induced more fear reactions in C-O. The reactions of Japanese quail chicks to humans depend on their mother’s level of habituation [45]. Obviously, elder mothers were more habituated to humans because they had spent longer in captivity and therefore their chicks should fear humans less. As we observed the contrary, we hypothesized that C-O’s fear reactions could be related to a higher level of global reactivity. This is supported by latencies to emerge into a novel environment, as observed in emergence or runway tests. Analyses of chicks’ sociality revealed no significant differences between the two sets concerning inter-individual distances or times spent in different zones in the runway test. Nevertheless, chicks of elder females were more reactive to social isolation. When separated from siblings, they moved less and tended to emit more distress calls than did chicks brooded by young females that expressed more comfort behaviours such as preening or exploring the ground. This higher reactivity of C-O to social isolation was also evidenced in an unfamiliar environment, the open-field, where they emitted more distress calls.

Experience with their foster mother is obviously at the root of chicks’ behavioural differences. Mother birds are known to influence offspring behavioural development but, to our knowledge, this influence has never been associated directly to mother-chick interactions during the breeding period, except for food preference [46]–[48]. Here we give the first illustration of the direct impact of variations of a bird’s maternal care on offspring behavioural characteristics as revealed by several tests. Our results agree with those of similar studies of mammals. Indeed, the fact that maternal rejection by primates can induce the development of a less anxious personality [23] can be compared to the lesser fearfulness of chicks exposed to greater maternal rejection.

Two main mechanisms are hypothesized to be involved in non-genomic influences of maternal care. The first mechanism consists in non-genetic social influences via different processes (imitation, social facilitation, local enhancement) [49]. This mechanism in particular is likely to concern Japanese quail as their chicks are capable of learning from their first post-hatch days [32] and mothers are a model for learning traits such as reactions to humans [45] or food preferences [46], [47]. The strength of this maternal influence could be related to the strength of the filial bond established between C-O and C-Y chicks with their mothers. Indeed, maternal behaviour has been reported to facilitate and to stimulate the development of filial imprinting [50]. By expressing more maternal behaviour, particularly at the beginning of the breeding period, elder females may have established a stronger filial imprinting in their chicks. A stronger imprinting in C-O might explain why they stayed close to the mothers for longer, and might be at the root of their greater reactivity, because a stronger social bond with their mother may enhance maternal influence and probably increase the effects of separation from their mother.

The second mechanism considers changes in the D.N.A. compaction state provoked by tactile stimulations. One of the most striking examples of this mechanism reveals that high levels of licking and grooming by mother rats induce epigenetic modifications of promoters for estrogen receptors in specific tissues in pups that will induce important licking and grooming of their own offspring when pup females become adult [22]. This kind of mechanism is particularly likely to occur in altricial species in which tactile stimulations are the most important component of the sensitive environment of young, but this mechanism could also occur in precocial birds via maternal warming.

To understand entirely the links between age and maternal influence, we must compare our postnatal observations to results concerning prenatal influences. Prenatal effects on both laying rates and chicks’ weight at hatching are consistent with the effort hypothesis; a previous study reported that older females had lower laying rates but that their chicks were heavier at hatching [31]. Results concerning prenatal effects on chicks’ behaviour appear very different from postnatal effects in that chicks from eggs laid by young females showed higher fear levels and lower reactivity to social isolation than did elder females’ chicks. Actually, when considered all together these results do not appear incoherent. The fact that young mothers’ chicks are less social could play a part in their earlier emancipation and their higher level of fearfulness could be related to lower investment by young mothers in brood defence. Indeed, although we did not evaluate brood defence in this study, so as to limit disturbing the brood, it increases with age in another gallinaceous, the red jungle fowl [19]. If young mothers are less capable of defending their brood, chicks must obviously be more reactive so as to increase their chances of survival when encountering a predator.

Our findings evidenced that elder females are more invested in brood care than are young females, as far as postnatal care is concerned. As our procedure focused on this particular part of the female’s reproduction behaviour, this implied standardizing conditions for the rest of the cycle, but age-related differences in mating, copulation or incubation should also be investigated under such conditions. The fact that maternal performance improved with age when breeding experiences were the same does not mean that maternal experience is not also involved in the increasing of offspring survival rates with mothers’ age. Consequently, maternal behaviour should be compared between same-aged females with different maternal experience.

Our results confirm that Japanese quail is a great model to help expand our understanding of maternal behaviour in birds and that the way we evaluate maternal behaviour enables us to evidence interesting qualitative and quantitative variations in maternal behaviour.
